# A single bout of downhill running attenuates subsequent level running-induced fatigue

**DOI:** 10.1038/s41598-020-76008-2

**Published:** 2020-11-02

**Authors:** Claudio de Oliveira Assumpção, Renan Vieira Barreto, Leonardo Coelho Rabello de Lima, Adalgiso Coscrato Cardozo, Maria Imaculada de Lima Montebelo, Helen Reinhart Camargo Catarino, Camila Coelho Greco, Benedito Sergio Denadai

**Affiliations:** 1 Human Performance Laboratory, Department of Physical Education, IB, São Paulo State University, UNESP, Av. 24A, 1.515, Bela Vista, Rio Claro, São Paulo CEP 13506-900 Brazil; 2 Biomechanics Laboratory, Department of Physical Education, São Paulo State University, Rio Claro, São Paulo Brazil; 3 Graduate Program in Human Movement Sciences, Methodist University of Piracicaba - UNIMEP, Piracicaba, São Paulo Brazil; 4 University Center of Itapira - UNIESI, Itapira, São Paulo Brazil

**Keywords:** Physiology, Metabolism, Homeostasis

## Abstract

Fatigue can be defined as exercise-induced strength loss. During running, fatigue can be partially explained by repetitive low-intensity eccentric contractions-induced muscle damage (EIMD). Previous studies showed that a bout of downhill running (DR) attenuated subsequent EIMD. Thus, we tested if a 30-min DR bout would attenuate fatigue induced by subsequent 60-min level running (LR). Twenty-seven male college students were randomly allocated to an experimental (EXP) or a control (CON) group. All participants performed LR on a treadmill at 70% of the velocity (vVO_2_peak) corresponding to peak oxygen uptake (VO_2_peak). Only EXP performed a 30-min DR (− 15%) on a treadmill at 70% vVO_2_peak fourteen days before LR. Indirect EIMD markers and neuromuscular function were assessed before, immediately and 48 h after DR and LR. Knee extension isometric peak torque (IPT) decreased (− 36.3 ± 26%, *p* < 0.05) immediately following DR with full recovery reached 48 h post-DR. Muscle soreness developed (*p* < 0.05) immediately (37 ± 25 mm) and 48 h (45 ± 26 mm) post-DR. IPT and rate of torque development (RTD) at late phases (> 150 ms) from the onset of muscle contraction decreased significantly (− 10.7 ± 6.1% and from − 15.4 to − 18.7%, respectively) immediately after LR for the CON group and remained below baseline values (− 5.6 ± 8.5% and from − 13.8 to − 14.9%, respectively) 48 h post-LR. However, IPT and late RTD were not significantly affected by LR for the EXP group, showing a group x time interaction effect. We concluded that a single DR bout can be used to attenuate fatigue induced by a LR performed fourteen days after.

## Introduction

Fatigue can be defined as a reversible decline in maximal force-generating capacity^1,2^. In recreational or high-level sports activities, excessive fatigue development may compromise physical performance and increase susceptibility to injuries^3^. This impairment in muscle function depends on a variety of factors related to the exercise such as type of contraction, muscle groups involved, and exercise intensity and duration. For instance, running seems to provoke considerable strength loss due to a large number of eccentric and concentric contractions of the knee extensor muscles^4^, which can alter intramuscular metabolite accumulation and motor unit recruitment^5^ while also damaging muscle fibers^6,7^.

Indeed, intense and/or repetitive eccentric contractions are well-known to induce ultrastructural disruption in skeletal muscle fibers, termed as exercise-induced muscle damage (EIMD), which is often quantified by the manifestation of delayed-onset muscle soreness (DOMS), increased plasma creatine kinase (CK), and strength loss^6,7^. However, after being exposed to EIMD, affected muscles become less susceptible to damage induced by a subsequent similar exercise, a phenomenon known as the repeated bout effect (RBE)^8^.

Previous studies have shown attenuation of EIMD symptoms (i.e. RBE) after a single session of downhill running (DR)^9–11^. Chen et al.^10^ showed that a 30-min DR bout at 70% VO_2_peak attenuated EIMD symptoms when subjects performed an identical DR bout one week before. Similarly, Smith et al.^11^ found a significant reduction in DOMS (~ 42%) and CK (~ 53%) activity after repeated bouts of 60-min DR at 75% VO_2_peak. Eston et al.^9^ demonstrated that five 8-min DR bouts at 7 miles/h separated by a 2-min rest interval attenuated changes in indirect markers of EIMD following a subsequent DR performed 5 weeks later. Although there is evidence that the RBE extends to exercise bouts with similar motions^8^, it is still unclear if the protective effect conferred by a bout of DR can attenuate EIMD and fatigue following an extensive bout of level running.

Therefore, since the strength loss induced by running exercise can be partially due to EIMD, and a single bout of DR confers a significant protective effect against subsequent EIMD, we tested the hypothesis that fatigue induced by level running exercise can be attenuated by previous DR-induced muscle damage. However, Peñailillo et al.^12^ verified that rate of torque development (RTD) at late phases (> 100 ms) from the onset of muscle contraction is a more specific and sensitive indirect marker of EIMD than maximal voluntary contraction (MVC). Thus, the purpose of this study was to investigate the efficacy of performing a 30-min DR bout on the attenuation of changes in both MVC and RFD of the knee extensors, immediately after and 48 h following a 60-min level run.

## Results

The characteristics of CON and EXP groups are expressed in Table 1. No significant differences in age, body mass, height, VO_2_peak, vVO_2_peak, and VO_2_ at the lactate threshold (LT) were found between groups at baseline.

**Table 1:** Mean (SD) of physical and physiological characteristics of control (CON) and experimental (EXP) groups.

	CON (N = 14)	EXP (N = 13)
Age (years)	24.6 ± 3.3	22.7 ± 4.4
Body mass (kg)	74.8 ± 10.9	72.8 ± 9.7
Height (cm)	175.5 ± 6.6	173.2 ± 7.0
VO_2_peak (mL kg min^−1^)	49.0 ± 6.9	49.9 ± 5.8
vVO_2_peak (km h^−1^)	16.0 ± 1.5	16.0 ± 2.2
VO_2_ at LT (ml kg min^−1^)	27 ± 4.8	27.9 ± 4.9
VO_2_ at LT (%VO_2_peak)	56.3 ± 13.3	56.1 ± 12.7

VO_2_peak: maximal oxygen uptake; vVO_2_peak: velocity at which the maximal oxygen uptake was attained; VO_2_: oxygen uptake; LT: lactate threshold.

### Downhill run

Baseline values for IPT, *vastus lateralis* EMG-RMS during IPT, serum CK activity and quadriceps muscle soreness before DR were 272 ± 46 N m, 115 ± 93%, 115 ± 93 U l^−1^, and 0.9 ± 1.3 mm, respectively, in EXP group.

**Figure 1:**
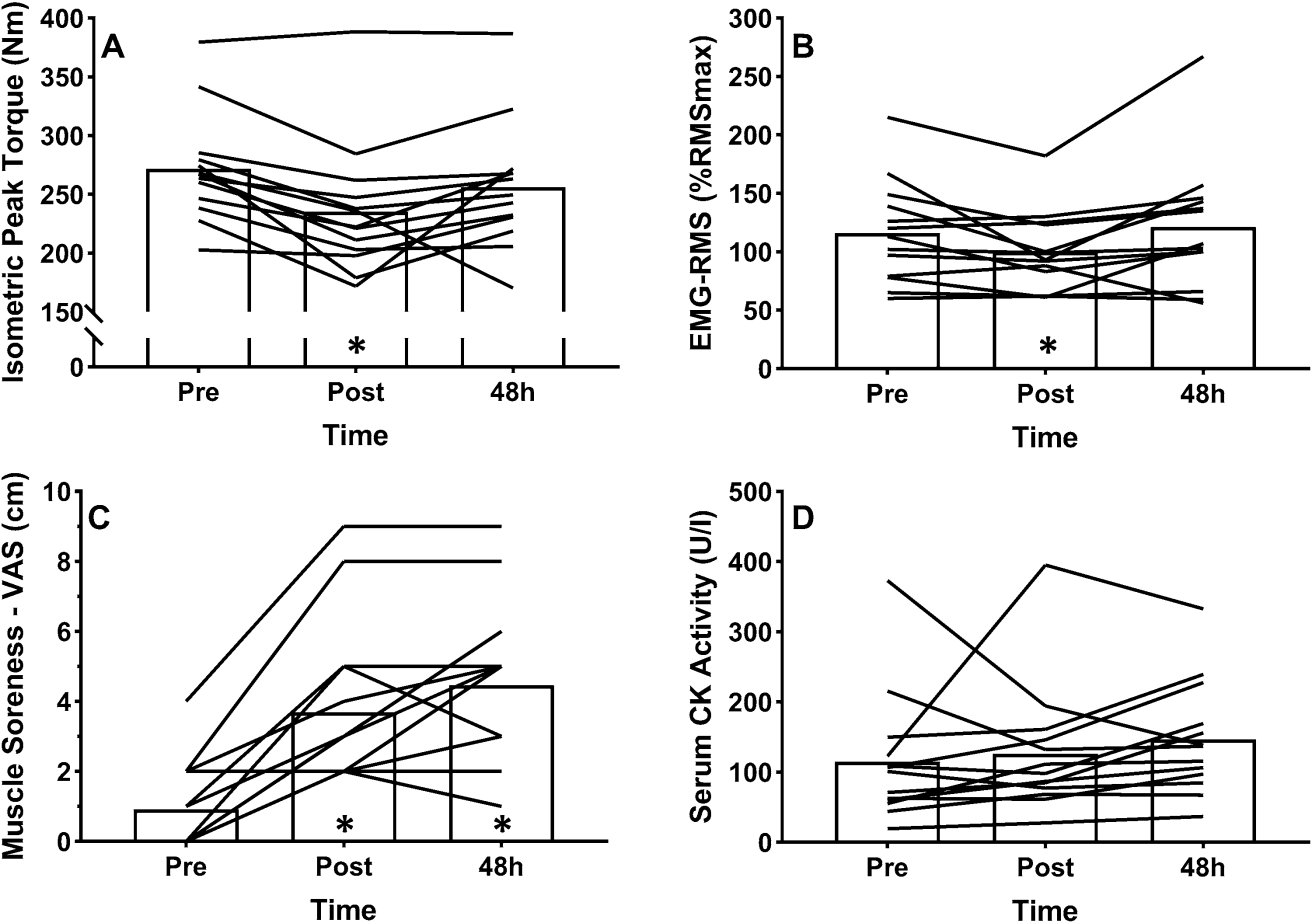
Changes in (**A**) isometric peak torque, (**B**) vastus lateralis EMG-RMS during isometric peak torque, (**C**) quadriceps muscle soreness, and (**D**) serum creatine kinase (CK) activity following downhill run for experimental group. *p < 0.05 compared to pre-DR.

Changes in neuromuscular function and indirect markers of EIMD following DR are expressed in Fig. 1. Significant effects of time were observed for IPT (F = 7.53, *p* = 0.003, η_p_^2^ = 0.38), *vastus lateralis* EMG-RMS during IPT (F = 4.99, *p* = 0.015, η_p_^2^ = 0.29) and quadriceps muscle soreness (F = 24.22, *p* < 0.001, η_p_^2^ = 0.67) following DR. Post hoc analyses showed a significant (− 36.3 ± 26%, *p* < 0.05) decrease of IPT immediately following DR with full recovery reached 48 h post-DR. EMG-RMS during IPT decreased significantly (*p* < 0.05) from 116 ± 44%RMSmax to 100 ± 34%RMSmax following DR and returned to baseline (121 ± 55%RMSmax) 48 h post-DR. Quadriceps muscle soreness developed immediately (3.7 ± 2.5 cm) and 48 h (4.5 ± 2.6 cm) post-DR. No significant effect of time was observed for serum CK activity (F = 0.89, *p* = 0.42, η_p_^2^ = 0.07).

### Level run

**Figure 2:**
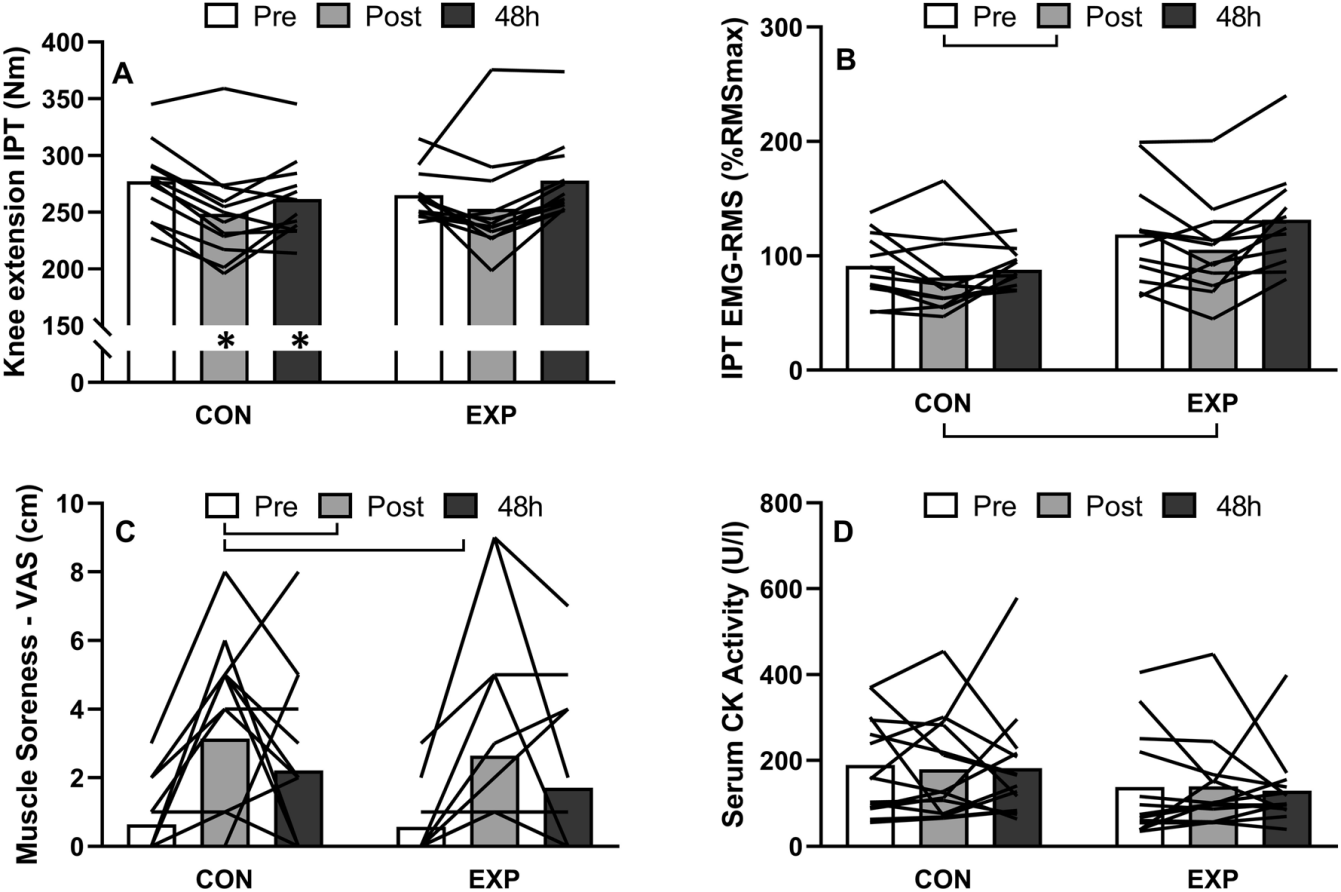
Changes in (**A**) knee extension isometric peak torque (IPT), (**B**) *vastus lateralis* EMG-RMS during isometric peak torque, (**C**) knee extensors muscle soreness, and (**D**) serum creatine kinase (CK) activity over time following 60-min of level run for control (CON) and experimental (EXP) groups. Brackets represent significant main effect of time; *p < 0.05 compared to baseline values for the same group. Analyses of variance of neuromuscular data (**A**,**B**) were performed with n = 12 for both groups due to missed assessment points.

Changes in IPT, EMG-RMS during IPT and indirect markers of EIMD are expressed in Fig. 2. A significant time × group interaction effect was found for IPT (F = 4.34, *p* = 0.02, η_p_^2^ = 0.16), which decreased significantly (− 10.7 ± 6.1%, *p* < 0.05) immediately after LR for the CON group and remained below baseline values (− 5.6 ± 8.5%, *p* < 0.05) 48 h post-LR. IPT was not significantly affected by LR for the EXP group. No significant difference between groups was found for IPT at any time point.

Significant effects of time were found for muscle soreness (F = 10.38, *p* < 0.001, η_p_^2^ = 0.32) and EMG-RMS during IPT (F = 5.36, *p* = 0.008, η_p_^2^ = 0.19) and a significant effect of group was found for EMG-RMS during IPT (F = 5.57 *p* = 0.03, η_p_^2^ = 0.20). Post hoc analyses revealed significant (*p* < 0.05) changes in EMG-RMS during IPT only immediately following LR. Muscle soreness developed immediately following LR (CON: 3.1 ± 2.7 cm; EXP: 2.5 ± 3.2 cm) and remained elevated until 48 h after it (CON: 2.2 ± 2.5 cm; EXP: 1.8 ± 2.4 cm). Analyses of variance did not result in significant effects of time (F = 0.11, *p* = 0.90, η_p_^2^ = 0.004), group (F = 3.01, *p* = 0.09, η_p_^2^ = 0.12), and group x time interaction (F = 0.01, *p* = 0.99, η_p_^2^ = 0.0004) for serum CK activity.

**Figure 3:**
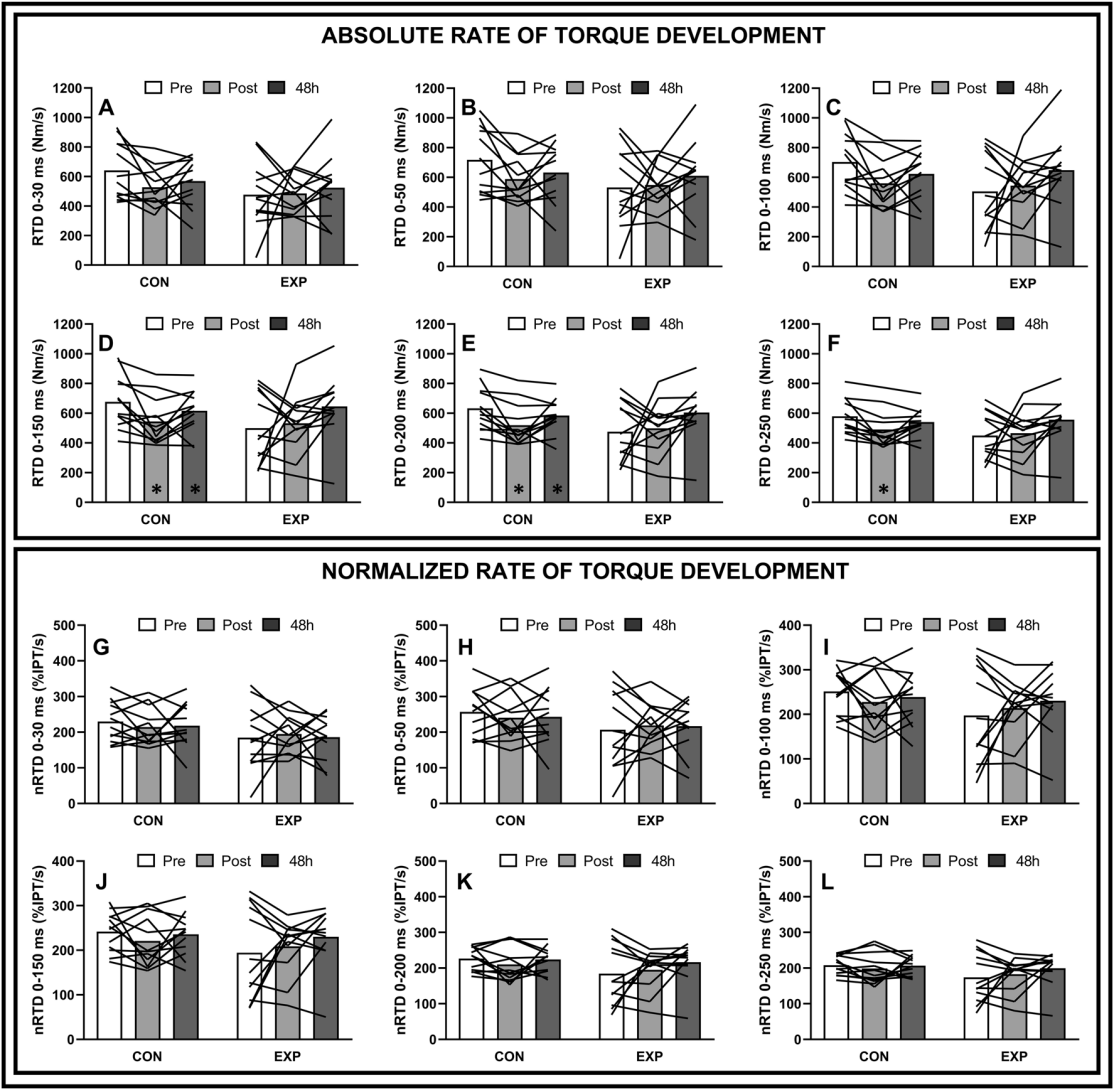
Changes in RTD and normalized RTD, respectively, achieved at 30 ms (**A**,**G**), 50 ms (**B**,**H**), 100 ms (**C**,**I**). 150 ms (**D**,**J**), 200 ms (**E**,**K**) and 250 ms (**F**,**L**) over time following 60-min of level run for control (CON) and experimental (EXP) groups. *p < 0.05 compared to baseline values for the same group. Analyses of variance were performed with n = 12 for both groups due to missed assessment points.

Changes in RTD and normalized RTD obtained at 0–30, 0–50, 0–100, 0–150, 0–200 and 0–250 ms are expressed in Fig. 3. Analyses of RTD achieved at 0–30, 0–50 and 0–100 ms resulted in no significant (*p* > 0.05) effects of time, group, or group × time interaction. Significant group x time interactions were found for RTD at 0–150 ms (F = 3.37, *p* = 0.04, η_p_^2^ = 0.13), 0–200 ms (F = 3.41, *p* = 0.04, η_p_^2^ = 0.13) and 0–250 ms (F = 3.21, *p* = 0.05, η_p_^2^ = 0.13). Pairwise analyses for the CON group revealed that RTD at 0–150 ms and 0–200 ms decreased (*p* < 0.05) immediately following LR (0–150: 17.6 ± 18,7%; 0–200: 16.9 ± 15.4%) and remained below baseline values following 48 h (0–150: 7.8 ± 12.3%; 0–200: 6.6 ± 10%) and that RTD calculated at 0–250 decreased (*p* < 0.05) immediately following LR (15.1 ± 12.7%), but returned to baseline values following 48 h. No significant changes in RTD were observed for the EXP group following LR. No significant difference between groups was found for RTD measured at any interval at any time point. No significant effects of time, group, and group x time interaction were found for normalized RTD at any of the calculated intervals.

## Discussion

The main original finding of this study was an attenuation of changes in both MVC and late RFD immediately after and 48 h following a 60-min LR when participants performed a single bout of DR 14 days before, which confirmed our hypothesis. We have suggested that prior DR would induce a protective effect against EIMD, which is one of the factors that contribute to the decreased MVC and late RFD observed immediately after and 48 h following running exercises^4^.

### Downhill running-induced muscle damage

The protective effect observed, known as RBE, consists of attenuation of EIMD after a first exposure to a damaging exercise^8^. In the present study, a significant reduction in IPT (immediately after) and development of muscle soreness (until 48 h) after the DR bout indicates the existence of EIMD corroborating with previous studies that employed this DR protocol^10,13,14^. Muscle soreness reported in the present study should, however, be interpreted with caution since self-palpation might have resulted in different pressures applied by each participant as well as by the same participant at different time points and this is a limitation of our study. Nevertheless, the magnitude of muscle soreness (35% increase) reported here was similar to that found in previous studies (~ 36% increase)^10,13^. However, the studies of Chen and colleagues^10,13,14^ also showed a significant increase in serum CK activity in addition to force loss and soreness after DR. Moreover, recovery of maximal isometric strength capacity reported in the aforementioned studies^10,13,14^ was slower (lasting for 5–7 days) than that reported in the present investigation (fully recovered 2 days post-DR) following the initial DR bout. These differences may be attributed to the recruitment of different populations among studies. For instance, the mean vVO_2_peak reported in the present study was 16 km h^−1^ whereas in Chen et al.^10^ was ~ 12 km h^−1^. This indicates that participants who volunteered for the present investigation may be more physically active than those who participated in the Chen et al.^10^ study, and, possibly, less susceptible to EIMD.

### Level running-induced fatigue

In the present study, the LR was performed at 70% vVO_2_peak and lasted for one hour, with the individuals of EXP group presenting lower magnitude of strength loss immediately after and 2 days following the exercise. The magnitude of strength loss found in the present study (~ 8%) is coherent with previously shown by others^15,16^ In the case of Oliveira et al.^16^, a reduction of ~ 5.7% in maximal isometric strength of knee extensors was reported after ~ 36 min of level run at ~ 70% vVO_2_max (8.5–9.0 km h^−1^). Millet et al.^15^ reported a decrease of 23.5% in IPT after a 30-km trial of self-paced running exercise that lasted about 2–3 h. Taken together, these results showed changes in isometric strength capacities that are proportional to the respective exercises loads, and our finding corroborate with such a notion.

In accordance with the literature, the LR was performed in the heavy intensity domain (i.e., above the LT), which involves exercises with duration between ~ 40 min and 3 h^17,18^. The impairment of neuromuscular function at these exercise intensities can be explained by central and peripheral mechanisms such as decreased neural drive, compromised motoneuron excitability and failure in excitation–contraction coupling, among others^19^. Specific alterations occurring within the central nervous system involved in the strength loss within the heavy intensity domain have not yet been fully understood^19^. However, studies have shown a greater reduction in voluntary activation when compared to the severe intensity domain^20,21^. Thus, the decrease in cortical output during heavy intensity exercise can contribute to the reduced capacity to produce force.

In the present study, a significant effect of time was observed for vastus lateralis EMG-RMS, suggesting a participation of central mechanism contributing to the strength loss. However, for the maximal EMG signal to be considered a central fatigue indicator, it is necessary to normalize the EMG-RMS value by the maximum M-wave (i.e., an indicator of neuromuscular propagation) to provide an index of muscle activation that is not influenced by changes in muscle excitability^4^. In fact, similar reductions in both M-wave and RMS were demonstrated following cycling lasting 2-h^22^ and level running lasting 45 min^23^, providing no evidence of the contribution of central mechanisms to fatigue. However, our experimental design (using only RMS value) does not allow differentiation of the origin of neuromuscular fatigue. Nevertheless, there was no effect of time and group x time interaction for early RTD (< 100 ms), which is mainly determined by both muscle activation, as assessed by surface EMG, and intrinsic muscle contractile properties^24,25^. Moreover, normalized RTD was not modified after LR (immediately and post 48 h) for both groups. Therefore, it is possible to hypothesize that the factors that determined the lower strength loss after LR can be predominantly related to peripheral factors. Peripherally, the strength loss during exercise performed within the heavy intensity domain may be related to depletion of glycogen stores, and to increased production of reactive nitrogen and oxygen species, which seem to interfere with Ca^2+^ release (for details, please see Brownstein et al.^19^). Additionally, in running, which involves the stretching-shortening cycle, the myofibrillar disintegration and the disorganization of sarcomeres generated by muscle damage can also contribute to the strength loss observed in the present study, especially 48 h post-LR^26^.

### Attenuation of strength loss and the repeated bout effect

Different magnitudes of strength loss and late RFD impairment following LR observed between groups in the present study are probably due to the RBE. Several mechanisms including neural and mechanical adaptations, extracellular matrix remodeling, and alteration in the inflammatory response are involved in the attenuation of subsequent EIMD^8^. In the present study, the DR protocol was performed 14 d before LR, thus, we assume that the adaptations that explain the RBE in this study lasted this period based on previous reports that DR-induced RBE lasts from 5 to 35 days^9–11^. Some mechanisms such as neural adaptations, which contributes to early manifestations (lasting in ~ 7 days) of the RBE, may not explain the observed protective effect^27^. Instead, adaptations such as extracellular matrix remodeling and changes in muscle–tendon complex behavior during LR may explain the attenuation of strength loss observed for EXP immediately after LR^28,29^. Such adaptations might have protected myofibers from severe mechanical strain through increased tendon compliance and/or increased passive tension by strengthening the extracellular matrix and therefore reducing EIMD during eccentric contractions.

Moreover, we found an attenuation of strength loss 48 h after LR. Strength loss observed immediately following long distance running is multifactorial and may be attributed to the mechanisms discussed in the section above as well as tissue damage and compromised excitation–contraction coupling caused by eccentric contractions. Long-lasting strength loss and reduced late RTD, such as that observed 48 h post-LR for the CON group, appears to be related to EIMD^6,7,12^. Hence, the absence of changes in both IPT and late RTD observed immediately and 48 h after LR for the EXP suggest that DR conferred significant protection against LR-induced muscle damage. The protective effect conferred by DR was not observed for serum CK activity and quadriceps muscle soreness, which also are indirect markers of EIMD. Although extensively used as a surrogate of EIMD, blood CK activity does not appear to be as influenced by running as it is by other exercise modalities that result in noticeably greater amounts of mechanical stress to eccentrically contracting muscle fibers^6,7,10–14^. This is corroborated by the fact that changes in serum CK activity failed to reach statistical significance following all LR conditions as well as DR in the present study. The same did not occur for muscle soreness, which developed similarly following LR for the EXP and CON groups, in contrast with IPT. However, as already stated, assessment of muscle soreness through self-palpation is a limitation of the present study, since the level of muscle soreness is negatively correlated with the level of pressure applied to the muscles. Moreover, data from Damas et al.^30^ showed that changes in maximal strength (i.e., IPT) are the main indirect markers of EIMD. Therefore, it is likely that changes in tendon compliance, extracellular matrix cohesion and inflammatory response (responsible for the attenuation of secondary muscle damage^31,32^) caused by the RBE have influenced the protective effect observed over strength loss following LR.

Previous studies that investigated strategies to attenuate muscle fatigue induced by LR showed different levels of attenuation of strength loss^16,33^. Easthope et al.^33^ investigated the RBE conferred by four bouts of trail running (~ 15.6 km) separated by seven days in active male runners. They found a progressive attenuation of running-induced fatigue from ~ 18% of isometric strength loss after the first bout to ~ 3% after the fourth bout^33^. Oliveira et al.^16^ showed a ~ 3% reduction in isometric strength loss induced by running after 8 weeks of an isokinetic eccentric resistance training protocol. Nevertheless, eccentric strength loss after a running protocol was reduced by ~ 11% following training^16^. However, the interventions used in these studies were longer (4–8 weeks of exercise) than that (a single DR bout) used in the present study.

As limitations, we acknowledge that the fatiguing effect of running exercise is contraction-type dependent, with greater loss of muscle function observed in dynamic eccentric contractions^16,34,35^. However, since the present study investigated the protective effects of DR on subsequent running exercise, eccentric contractions were avoided during muscle function assessments to prevent the occurrence of undesired EIMD and/or the manifestation of a RBE.

## Conclusions

To our knowledge, this is the first study investigating the efficacy of previous DR on attenuation of level running-induced fatigue. We conclude that a single bout of 30-min DR can be used to reduce strength loss after long term LR performed 14 after. This strategy can be useful to minimize the risk of injuries related to running-induced strength loss. Moreover, an attenuation of fatigue induced by running exercises may be interesting to maintain physical performance in contexts where different exercise modalities are performed consecutively such as combined training programs that usually include running followed by resistance exercises.

## Methods

### Participants

Twenty-seven male college students participated in this study, after reading and signing a consent form. They were allocated to either an experimental (EXP; N = 13) or a control (CON; N = 14) group in a counterbalanced randomized fashion by drawing the group for every odd participant and allocating even participants to the opposite group. All subjects were asked to maintain their regular diets and routines and not to take any non-steroidal anti-inflammatory drugs or analgesic drugs during the entire experiment. None of the subjects had prior experience with systematic resistance or endurance training in the 6 months preceding the experimental period. All risks associated with the experimental procedures were explained to the subjects prior to study participation, and each participant signed an informed consent form. The study protocol is in accordance with the Declaration of Helsinki on the use of humans as research subjects and was approved by the local Ethics Committee of São Paulo State University (Protocol n^o^ 101/2011).

### Experimental design

All participants performed a 60-min level run (LR) on a treadmill with 1% slope at a speed of 70% of the velocity corresponding to VO_2_peak (vVO_2_peak) (CON: 11.2 ± 1 km h^−1^; EXP: 11.2 ± 1.5 km h^−1^. Neuromuscular function (NMF) and indirect markers of EIMD were assessed before, immediately after and 48 h after LR. Fourteen days before LR, subjects in the EXP group performed a 30-min DR on a treadmill with a slope of − 15% at 70% vVO_2_peak^10^ (11.2 ± 1.5) while subjects in CON rested. This DR protocol has been adopted previously^10,12,14^ and proven to confer a potent RBE^10^. Indirect markers of EIMD and NMF were assessed before, immediately and 48 h after DR.

**Figure 4:**
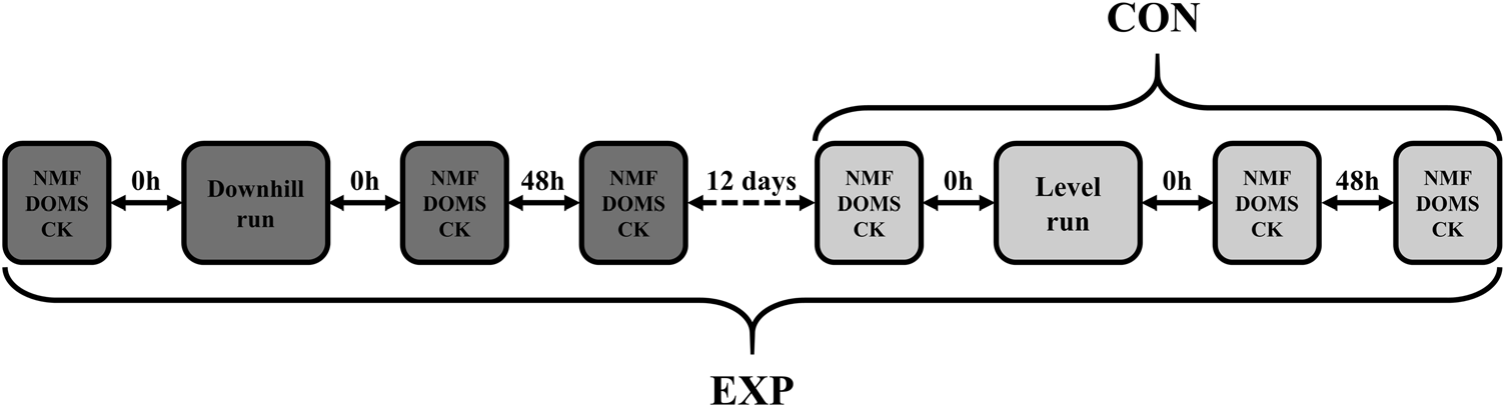
Experimental design of the study. Dark-grey boxes represent assessments and intervention of experimental group (EXP) while light-grey boxes stand for assessments and intervention of both EXP and control (CON) groups. *NMF* neuromuscular function, *DOMS* delayed-onset muscle soreness, *CK* serum creatine kinases activity.

Before the experimental session, participants visited the laboratory to undergo a familiarization session. In this occasion, they performed ten submaximal and three maximal voluntary isometric contractions with the knee extensors. The maximal voluntary isometric contractions were identical to the contractions performed to assess NMF. In this occasion, participants were also trained to assess knee extensor muscle soreness via self-palpation. Lactate threshold (LT) and VO_2_peak were determined following familiarization procedures. The familiarization session was conducted 5–7 days prior to the first experimental session for both groups. Figure 4 illustrates the experimental design.

### Lactate threshold

Initially, each subject performed a submaximal step incremental test to determine the LT. The test started at 4 km h^−1^ and was increased by 1 km h^−1^ every 3 min for four to five stages. Capillary blood samples were collected within the final 20 s of each stage for [La] determination. The LT was determined from the relationship between [La] and the walking/running speed and was considered as the first sudden and sustained increase in [La] above resting concentration^36^.

### Peak oxygen uptake

VO_2_peak was determined using an incremental ramp test starting at 7 km h^−1^ with continuous increases of 1 km h^−1^ per minute (~ 0.16 km h^−1^ each second) until volitional exhaustion. Participants’ gas exchanges were monitored breath-by-breath using a gas analyzer (Quark PFT Ergo, Cosmed, Italy) throughout the entire test. The obtained oxygen uptake values were filtered using 15-s windows. VO_2_peak was calculated as the greatest oxygen uptake value obtained during the test following filtering and the speed at which it occurred (vVO_2_peak) was registered for further analyses.

### Criterion measures

#### Neuromuscular function

Due to technical issues at different time points, the sample size for neuromuscular data was 12 participants per group. Knee extensors isometric peak torque (IPT) was assessed at a knee joint angle of 75° on an isokinetic dynamometer (System 3, Biodex Systems, USA) connected to an acquisition module (Miotool 200/400, Brazil) with a capture frequency of 1000 Hz. Participants were seated and securely strapped onto the dynamometer chair. Two 5-s maximal voluntary isometric contractions were performed with a 3-min rest interval in between. Participants were instructed to extend their knees as fast and forcefully as possible during each contraction. Torque data was filtered (Butterworth filter, low pass, 4th order with a 15 Hz cut-off frequency) and stored for further analyses in MatLab (MatLab 6.5, Mathworks, USA). IPT was calculated as the greatest torque value during maximal voluntary isometric contraction. Rate of torque development (RTD) was calculated for the contraction with the greatest IPT as the slope of the torque-time curve at fixed intervals between the onset of the contraction (0 ms) and 30, 50, 100, 150, 200 and 250 ms. The onset of muscle contraction was considered as the point in time when torque values exceeded 2.5% of the difference between baseline and peak torque values^37^. Normalized RTD was calculated at the same time intervals by dividing RTD by IPT.

Electromyographic (EMG) signal of the *vastus lateralis* muscles was assessed during maximal voluntary isometric contractions via disposable Ag/AgCl bipolar electrodes positioned over the skin surface of the muscle according to SENIAM recommendations for electrode placement^38^. Electrode positioning was carefully reproduced during every assessment by using reference points such as moles, scars and anatomical reference points. A reference electrode was placed over the ulnar styloid process. Prior to electrode placement, the skin was shaved, abraded and cleansed with alcohol. Electrodes were connected to a preamplifier (100 times gain) and to a biological signal acquisition module (20 times gain) (Miotool 200/400, Brazil). EMG analyses were performed in MatLab (MatLab 6.5, Mathworks, USA). High pass (Butterworth, high pass, 2nd order, with 20 Hz cut-off frequency) and low pass (Butterworth, low pass, 4th order, with 500 Hz cut-off frequency) filters were applied. Average root mean square (RMS) of the EMG signal obtained between 0.25 s before and 0.25 s after the IPT was normalized by the RMS of the EMG signal obtained at the same 0.5-s window of a reference maximal voluntary isometric contraction performed at the last day of the familiarization period in such a manner that all EMG-RMS values were expressed as % of RMSMax.

#### Creatine kinase activity

For the determination of serum creatine kinase activity (CK), 500 μl of blood were drawn from the antecubital vein by a trained nurse. Plasma was separated from serum in a centrifuge (CR-22-G, Hitachi, Japan) for 10 min at 2000 g. Serum was then analyzed in a spectrophotometer (Power Wave XS2, Biotek, Germany) with a commercial analysis kit (CK-NAC UV, Bioclin, Brazil) with normal reference values ranging from 95 to 195 (U l^−1^).

#### Muscle soreness

Quadriceps muscle soreness was determined via self-palpation. Participants were instructed to palpate their thighs by applying pressure to the anterior portion of the thigh with the thumbs at three distinct lengths: proximal, medial and distal. Following this, participants were instructed to rate perceived soreness in a 100-mm Visual Analog Scale (VAS) with “not sore at all” at one extremity and “very, very sore” at the other. Participants were trained to reproduce both the pressure and sites for palpation prior to the experiment. The rating was then measured with a ruler and recorded for further analyses.

### Statistical analyses

Based on IPT changes from before to immediately after LR at 70% vVO_2_peak in our previous study^16^, we estimated the required sample size as 12 participants with an alpha of 0.05 and a beta of 0.20 (i.e. power of 0.8). Data normality was tested by the Shapiro-Wilks test and the sphericity assumption was confirmed by Mauchly’s Sphericity test. Data is expressed as means ± SD unless otherwise stated. Anthropometric data, VO_2_peak, speed at VO_2_peak and VO_2_ at LT were compared between subjects with t-tests for unpaired samples. Changes in indirect markers of EIMD and neuromuscular function over time following DR were tested using repeated-measures one-way ANOVAs followed by Fisher’s post hoc tests when appropriate. Differences in neuromuscular function over time and between groups were tested using factorial two-way ANOVAs for repeated (time: 3 factors) and non-repeated (groups: 2 factors) followed by Fisher’s post hoc tests when significant effects of time or group were found. When a significant group *vs* time interaction was found, pairwise comparisons were performed by running one-way ANOVAs followed by Fisher’s post hoc tests for changes over time within in each group and Student’s t-tests for unpaired samples for differences in absolute values between groups. Effect sizes (partial eta squared, η_p_2) were calculated for the ANOVA main effects, and the magnitude of η_p_^2^ was classified as small ≤ 0.06, moderate 0.07–0.14 and large > 0.14^39^. Significance level was set as *p* ≤ 0.05. All analyses were performed using the Statistical Package for Social Sciences (SPSS 21.0, IBM, U.S.A.). Data are expressed as means ± SD of absolute values in figures and as percentage changes (%Pre) in the text.
